# Draft Genome Sequence of Salmonella enterica subsp. *enterica* Serovar Enteritidis ODA 99-30581-13, a Heat-Resistant Strain Isolated from Shell Eggs

**DOI:** 10.1128/MRA.01461-20

**Published:** 2021-03-04

**Authors:** Yumin Xu, Ahmed G. Abdelhamid, Ahmed E. Yousef

**Affiliations:** a Department of Food Science and Technology, The Ohio State University, Columbus, Ohio, USA; b Department of Microbiology, The Ohio State University, Columbus, Ohio, USA; University of Maryland School of Medicine

## Abstract

Salmonella enterica serovar Enteritidis ODA 99‐30581‐13 is a relatively heat-resistant strain isolated from shell eggs. The strain has a 4,777,965-bp genome sequence (52.1% GC content) that was predicted to encode 4,455 proteins, including heat stress response proteins and stress response regulators; these may be involved in its heat resistance.

## ANNOUNCEMENT

Salmonella enterica serovar Enteritidis has caused numerous salmonellosis outbreaks linked to egg consumption (1–4). Thermal pasteurization methods were developed to combat *Salmonella* contamination in shell eggs. Considering its relative heat resistance, Salmonella Enteritidis ODA 99‐30581‐13 was used as the target pathogen of shell egg pasteurization (5, 6). The current work presents the genome sequence of strain ODA 99‐30581‐13 and the genes likely linked to its heat resistance.

Strain ODA 99‐30581‐13 was streaked from frozen stock onto tryptic soy agar medium (BD, Sparks, MD), followed by culturing in tryptic soy broth (BD); incubation in both transfers was done at 37°C for 24 h. Genomic DNA (gDNA) was isolated from 1 ml of the overnight culture (10^9^ CFU/ml) using a QIAamp DNA minikit (Qiagen, Germantown, MD) according to the manufacturer’s protocol. The purity and concentration of the gDNA were evaluated by the NanoVue Plus spectrophotometer (Biochrom USA, Holliston, MA). DNA libraries of the gDNA were prepared using the Nextera DNA Flex library preparation kit (Illumina, Madison, WI) and barcoded using the Nextera DNA CD index library indexing kit (Illumina) according to the manufacturer’s instructions. The DNA libraries were quantified using the Qubit fluorimeter (Invitrogen, Waltham, MA), and their sizes were measured using the 2100 Bioanalyzer system (Agilent Technologies, CA). The genome was sequenced using the Illumina MiSeq platform (Food Microbiology Laboratory, The Ohio State University, Columbus, OH). The platform generated 478.5 Mb of data, including 1,910,230 raw reads (paired-end, 2 × 300-bp format) with 214.3× coverage and a quality score of 37, calculated by FastQC v0.11.9 software (7). Using Illumina’s “Generate FASTQ analysis” module to trim adaptor sequences and SPAdes v3.10.1 (8) for *de novo* assembly resulted in 30 contigs (*N*_50_, 491,928 bp), which were ordered against the reference genome of *Salmonella enterica* serovar Typhimurium LT2 by progressiveMauve v2.4.0 (9). Annotation of the *de novo*-assembled genome sequence was realized through the NCBI Prokaryotic Genome Annotation Pipeline (10). Default settings were applied for the bioinformatics software that was used in this study.

Analysis of the Salmonella Enteritidis ODA 99‐30581‐13 genome sequence showed that it has 4,777,965 bp (GC content, 52.1%) and encodes 4,455 proteins, 75 tRNAs, 15 rRNAs, and 15 noncoding RNAs (ncRNAs). Strain ODA 99‐30581‐13 belongs to sequence type 11 and serotype Enteritidis, as determined by MLST v2.0 (11) and SeqSero v1.2 (12), respectively. The genome of ODA 99‐30581‐13 harbors genes involved in heat shock response (*dnaK*, *dnaJ*, *grpE*, *clpP*, *hscAB*, *htrA*, *hsiJ*, *hspQ*, *hsp15*, *hsp20*, *ibpA*, *ibpB*, and *surA*) and heat stress response regulators (*rpoS* and *rpoH*) (13). Additionally, the general stress protein (NCBI Protein accession number WP_000807638.1; locus_tag IAF69_RS06740), which promotes heat resistance specific to Salmonella Enteritidis (14), was encoded in the ODA 99-30581-13 annotated genome sequence. The presence of these heat stress response-related genes might explain the relative heat resistance of strain ODA 99‐30581‐13.

### Data availability.

The genome sequence of Salmonella Enteritidis ODA 99‐30581‐13 was deposited at GenBank under accession number JACTGY000000000.1, BioProject accession number PRJNA659787, BioSample accession number SAMN15925966, and SRA accession number SRX9029726.
